# Supplementary material for: Is there an association between long-term antibiotics for acne and subsequent infection sequelae and antimicrobial resistance? A systematic review protocol
Source: BMJ Open. 2020 Jul 2;10(7):e033662. doi: 10.1136/bmjopen-2019-033662 (PMC7333805; doi:10.1136/bmjopen-2019-033662)
Supplement: Supplementary data [file bmjopen-2019-033662supp001.pdf]

Database: Ovid MEDLINE(R) and Epub Ahead of Print, In-Process & Other Non-Indexed Citations, Daily and Versions(R) <1946 to June 21, 2019>
Search Strategy:
----
1 acne.mp. (17491)
2 exp Acne Vulgaris/ (11259)
3 1 or 2 (17491)
4 antibiotic*.mp. (355427)
5 exp Antibiotic Prophylaxis/ (13110)
6 exp Anti-Bacterial Agents/ (700080)
7 tetracycline*.mp. (45045)
8 exp Tetracycline/ (19631)
9 exp Tetracyclines/ (46884)
10 lymecycline*.mp. (168)
11 exp Lymecycline/ (119)
12 minocycline*.mp. (8527)
13 exp Minocycline/ (5724)
14 doxycycline*.mp. (16071)
15 exp Doxycycline/ (9287)
16 oxytetracycline*.mp. (8262)
17 exp Oxytetracycline/ (6279)
18 macrolide*.mp. (22555)
19 Macrolides/ (11795)
20 exp Erythromycin/ (24397)
21 erythromycin*.mp. (25510)
22 clarithromycin*.mp. (10167)
23 exp Clarithromycin/ (6062)
24 azithromycin*.mp. (8538)
25 exp Azithromycin/ (4820)
26 dihydrofolate reductase inhibitor*.mp. (346)
27 exp Folic Acid Antagonists/ (57013)
28 trimethoprim*.mp. (21485)
29 exp Trimethoprim/ (11693)
30 exp Trimethoprim, Sulfamethoxazole Drug Combination/ (6696)
31 penicillin*.mp. (82869)
32 exp Penicillin-Binding Proteins/ (3293)
33 exp Penicillin G/ (38077)
34 cephalosporin*.mp. (32358)
35 exp Cephalosporins/ (41273)
36 exp beta-Lactamases/ (22172)
37 fluoroquinolone*.mp. (22199)
38 exp Fluoroquinolones/ (31393)
39 exp Ciprofloxacin/ (12824)
40 aminoglycoside*.mp. (23235)
41 exp Aminoglycosides/ (151256)
42 exp Gentamicins/ (18634)
43 antimicrobial*.mp. (154537)
44 exp Antimicrobial Stewardship/ (725)
45 exp Disk Diffusion Antimicrobial Tests/ (1536)
46 4 or 5 or 6 or 7 or 8 or 9 or 10 or 11 or 12 or 13 or 14 or 15 or 16 or 17 or 18 or 19 or 20

or 21 or 22 or 23 or 24 or 25 or 26 or 27 or 28 or 29 or 30 or 31 or 32 or 33 or 34 or 35 or 36 or 37 or 38 or 39 or 40 or 41 or 42 or 43 or 44 or 45 (1080981)
47 resistance*.mp. (827828)
48 exp beta-Lactam Resistance/ (26155)
49 exp Drug Resistance, Microbial/ or exp Microbial Sensitivity Tests/ (231349)
50 exp Drug Resistance, Multiple/ (33795)
51 exp Drug Resistance, Bacterial/ (83040)
52 exp Methicillin Resistance/ (10188)
53 exp Multidrug Resistance-Associated Proteins/ (14320)
54 exp Vancomycin Resistance/ (3263)
55 47 or 48 or 49 or 50 or 51 or 52 or 53 or 54 (900383)
56 43 or 44 [antimicrobial altogether] (154537)
57 55 and 56 [antimicrobial AND resistance] (70921)
58 46 and 55 [antibiotic AND resistance] (248811)
59 infect*.mp. (2131927)
60 exp Escherichia coli/ (270735)
61 exp Bacteriophages/ (56525)
62 exp Infection/ (760393)
63 infection*.mp. (1804659)
64 59 or 60 or 61 or 62 [infection altogether] (2649927)
65 55 or 57 or 58 [resistance OR antimicrobial resistance OR antibiotic resistance] (900383)
66 64 or 65 [infection OR resistance altogether] (3306493)
67 3 and 66 [combined with acne] (3142)
