# Supplementary material for: Is there an association between long-term antibiotics for acne and subsequent infection sequelae and antimicrobial resistance? A systematic review protocol
Source: BMJ Open. 2020 Jul 2;10(7):e033662. doi: 10.1136/bmjopen-2019-033662 (PMC7333805; doi:10.1136/bmjopen-2019-033662)
Supplement: Author's manuscript [file bmjopen-2019-033662.draft_revisions.pdf]

BMJ Open

BMJ Open is committed to open peer review. As part of this commitment we make the peer review history of every article we publish publicly available.

When an article is published we post the peer reviewers' comments and the authors' responses online. We also post the versions of the paper that were used during peer review. These are the versions that the peer review comments apply to.

The versions of the paper that follow are the versions that were submitted during the peer review process. They are not the versions of record or the final published versions. They should not be cited or distributed as the published version of this manuscript.

BMJ Open is an open access journal and the full, final, typeset and author-corrected version of record of the manuscript is available on our site with no access controls, subscription charges or pay-per-view fees (http://bmjopen.bmj.com).

If you have any questions on BMJ Open's open peer review process please email info.bmjopen@bmj.com

BMJ Open

Is there an association between long-term antibiotics for acne and subsequent infection sequelae and antimicrobial resistance? Systematic review protocol

Journal:	*BMJ Open*
Manuscript ID	bmjopen-2019-033662
Article Type:	Protocol
Date Submitted by the Author:	16-Oct-2019

Complete List of Authors:	Bhate, Ketaki; London School of Hygiene and Tropical Medicine Faculty of Epidemiology and Population Health,<br>Lin, Liang-Yu; London School of Hygiene and Tropical Medicine Faculty of Epidemiology and Population Health, Department of Non-communicable Disease Epidemiology;<br>Barbieri, John<br>Leyrat, Clémence; London School of Hygiene and Tropical Medicine, Department of Medical Statistics<br>Hopkins, Susan; Public Health England<br>Stabler, Richard<br>Shallcross, Laura; University College London<br>Smeeth, Liam; London School of Hygiene and Tropical Medicine Faculty of Epidemiology and Population Health<br>Francis, Nick A.; Cardiff University<br>Langan, Sinead; London School of Hygiene and Tropical Medicine<br>Sinnott, Sarah-Jo; London School of Hygiene and Tropical Medicine, Non-communicable disease epidemiology

Keywords:	Acne < DERMATOLOGY, antimicrobial resistance, EPIDEMIOLOGY

SCHOLARONE™
Manuscripts

I, the Submitting Author has the right to grant and does grant on behalf of all authors of the Work (as defined in the below author licence), an exclusive licence and/or a non-exclusive licence for contributions from authors who are: i) UK Crown employees; ii) where BMJ has agreed a CC-BY licence shall apply, and/or iii) in accordance with the terms applicable for US Federal Government officers or employees acting as part of their official duties; on a worldwide, perpetual, irrevocable, royalty-free basis to BMJ Publishing Group Ltd ("BMJ") its licensees and where the relevant Journal is co-owned by BMJ to the co-owners of the Journal, to publish the Work in this journal and any other BMJ products and to exploit all rights, as set out in our licence.

The Submitting Author accepts and understands that any supply made under these terms is made by BMJ to the Submitting Author unless you are acting as an employee on behalf of your employer or a postgraduate student of an affiliated institution which is paying any applicable article publishing charge ("APC") for Open Access articles. Where the Submitting Author wishes to make the Work available on an Open Access basis (and intends to pay the relevant APC), the terms of reuse of such Open Access shall be governed by a Creative Commons licence – details of these licences and which Creative Commons licence will apply to this Work are set out in our licence referred to above.

Other than as permitted in any relevant BMJ Author's Self Archiving Policies, I confirm this Work has not been accepted for publication elsewhere, is not being considered for publication elsewhere and does not duplicate material already published. I confirm all authors consent to publication of this Work and authorise the granting of this licence.

For peer review only

BMJ PROTOCOL

Title

Is there an association between long-term antibiotics for acne and subsequent infection sequelae and antimicrobial resistance? Systematic review protocol

Authors

Dr Ketaki Bhate[1] ksbhate@outlook.com https://orcid.org/0000-0001-5509-4428

Dr Liang-Yu Lin[1] liang-yu.lin@lshtm.ac.uk https://orcid.org/0000-0003-4720-6738

Dr John Barbieri[2] john.barbieri@uphs.upenn.edu https://orcid.org/0000-0002-5467-4102

Dr Clemence Leyrat[1] clemence.leyrat@lshtm.ac.uk https://orcid.org/0000-0002-4097-4577

Dr Susan Hopkins[2] susan.hopkins@phe.gov.uk https://orcid.org/0000-0001-5179-5702

Dr Richard Stabler[3] Richard.stabler@lshtm.ac.uk https://orcid.org/0000-0002-2402-6630

Dr Laura Shallcross[4] l.shallcross@ucl.ac.uk https://orcid.org/0000-0003-1713-2555

Professor Liam Smeeth[1] liam.smeeth@lshtm.ac.uk https://orcid.org/0000-0002-9168-6022

Professor Nick Francis[5] francisna@cardiff.ac.uk https://orcid.org/0000-0001-8939-7312

Professor Sinéad Langan[1*] sinead.langan@lshtm.ac.uk https://orcid.org/0000-0002-7022-7441

Dr Sarah-Jo Sinnott[1*] sarah-jo.sinnott@lshtm.ac.uk https://orcid.org/0000-0001-7586-686X

*Senior authors

Author affiliations

1. Faculty of Epidemiology and Population Health, London School of Hygiene and Tropical Medicine, Keppel Street, London.
2. University of Pennsylvania, Perelman School of Medicine, Philadelphia.
3. AMR Division, Public Health England.
4. Faculty of Infectious and Tropical Diseases, London School of Hygiene and Tropical Medicine, Keppel Street, London.
5. Faculty of Population Health Sciences, University College London.
6. School of Medicine, University of Cardiff.

For peer review only - http://bmjopen.bmj.com/site/about/guidelines.xhtml

Correspondence to: Dr Ketaki Bhate

London School of Hygiene and Tropical Medicine, Keppel Street, WC1E 7HT

Ketaki.bhate@lshtm.ac.uk

Word count

2060

Keywords

Acne vulgaris, antibiotic, antimicrobial resistance, tetracycline, macrolide, dihydrofolate reductase inhibitor

Author contributions

Ketaki Bhate wrote the protocol. Sinéad Langan and Sarah-Jo Sinnott supervised the writing process and contributed equally. Ling-Yu Lin, Clemence Leyrat, Richard Stabler, Laura Shallcross, Susan Hopkins, Nick Francis and Liam Smeeth form an advisory group and reviewed the protocol.

Funding statement

Ketaki Bhate is funded by an NIHR Doctoral Research Fellowship DRF-2018-11-ST2-066.

SML is funded by a Wellcome Trust Senior Clinical Fellowship (205039/Z/16/Z).

Dr. Barbieri is supported by the National Institute of Arthritis and Musculoskeletal and Skin Diseases of the National Institutes of Health under award number T32-AR-007465.

Disclaimer:

The views expressed are those of the author(s) and not necessarily those of the NHS, the NIHR or the Department of Health and Social Care.

For peer review only - http://bmjopen.bmj.com/site/about/guidelines.xhtml

Competing interests

John Barbieri receives partial salary support through a Pfizer Fellowship grant to the Trustees of the University of Pennsylvania.

For peer review only

For peer review only - http://bmjopen.bmj.com/site/about/guidelines.xhtml

ABSTRACT:

Introduction

Antimicrobial resistance (AMR) is a global health emergency. Acne vulgaris is a highly prevalent condition, and the dominant role antibiotics play in its treatment is a major concern. Antibiotics are widely used in the treatment of acne predominantly for their anti-inflammatory effect, hence their use in acne may not be optimal. Tetracyclines and macrolides are the two most common oral antibiotics classes prescribed, and average use can extend from –a few months to several years of intermittent or continuous use. This systematic review aims to elucidate what is known about oral antibiotics for acne contributing to AMR.

Methods and Analysis

A systematic review will be conducted to address the question: What is the existing evidence that long-term oral antibiotics used to treat acne in those over 8 years of age contribute towards increased infectious outcomes or other outcomes suggestive of the impact of AMR? We will search the following databases: Embase, MEDLINE, the Cochrane library and Web of Science. Search terms will be developed in collaboration with a librarian by identifying keywords from relevant articles and by undertaking pilot searches. Randomised-control trials, cohort and case-controlled studies conducted in any health care setting and published in any language will be included. The searches will be re-run prior to final analyses to capture the recent literature. The Cochrane tool for bias assessment in randomised trials and ROBINS-I for the assessment of bias in non-randomised studies will be used to assess the risk of bias of included studies. GRADE will be used to make an overall assessment of the quality of evidence.[1] A quantitative assessment will be undertaken of the outcome measures if the individual studies are sufficiently homogenous. If a quantitative assessment is not possible, a qualitative assessment will be presented as a narrative review.

Ethics and dissemination

Ethical approval is not required for this systematic-review. The results will be published in a peer-reviewed journal and any deviations from the protocol will be clearly documented in the published manuscript of the full systematic-review.

Prospero registration number

CRD42019121738.

For peer review only - http://bmjopen.bmj.com/site/about/guidelines.xhtml

Strengths and limitations of this study

- To our knowledge, this is the first comprehensive systematic review that will address the use of oral antibiotics for acne and their contribution to antimicrobial resistance
- Screening, data extraction and quality assessment will be undertaken independently by three medically qualified researchers with training in systematic review methodology, thereby ensuring scientific rigour, transparency and repeatability
- There are no date or language restrictions; however, this systematic review does not examine the grey literature

For peer review only

Introduction

The future effectiveness of antibiotics is in jeopardy with the World Health Organisation declaring the threat of Antimicrobial Resistance (AMR) a most urgent crisis. [2] Future deaths from infections as a result of AMR without any intervention is estimated at 10 million per year and by 2050, the cost of AMR could reach 100 trillion USD.[3]

Topical and oral antibiotics are commonly prescribed for the treatment of acne vulgaris, a chronic non-infectious skin disorder with onset predominantly in adolescence. Given the psychosocial consequences and potential for permanent disfigurement with scarring, it is imperative that people with acne receive effective treatment.[4, 5] Prevalence studies show that 80-100% of teenagers have acne and that 20% are moderately to severely affected. The high prevalence means that both topical and oral antibiotics are used in a large proportion of the adolescent population and for variable durations ranging from 6 weeks to many months, and in some cases, several years.[6, 7] Differences between international guidelines regarding duration of treatment is one of the reasons that antibiotics for acne are used for significantly longer than recommended as there is uncertainty about the optimal duration of treatment.[7-12] Tetracyclines and macrolides are the two of the most common oral antibiotic classes prescribed for acne with varying durations of average use depending on treatment setting and between different countries.[7, 13]

The overuse of antibiotics is a known cause of AMR as repeated and sustained exposure allows microbes to develop mechanisms to avoid the effects of the drugs designed to treat them and allows selection in favour of bystander or commensal bacteria with resistance subsequently cause invasive infection. Although acne is not an infectious disease and aetiologically is multifactorial, we already know that some strains of *Cutibacterium acnes* (formally *Propionibacterium acnes*), the bacteria pathophysiologically associated with acne, are now resistant to commonly used antibiotics in acne, making their initial use as anti-microbial agents futile.[14, 15] However, we do not know how these long-term antibiotics for acne may attenuate microbiota elsewhere at other body sites, and the ability of other bacteria at other infective sites to withstand the effect of antibiotics. Despite this, the anti-inflammatory effect and proven efficacy of antibiotics in treating acne ensures their continued use[16], albeit their effects may not be sustained. Considering the relationship between long term exposure to antibiotics and AMR, this practice may not be optimal.

The effects long-term antibiotics for acne have on future infections caused by resistant organisms, subsequent antibiotic treatment failure or the rate of infections (or any other measures which may indicate antimicrobial resistance) and how long any effect may last, is not yet known and has not been systematically reviewed in the literature before. While antibiotic stewardship programmes have been

For peer review only - http://bmjopen.bmj.com/site/about/guidelines.xhtml

shown to be effective [17] in other settings, to ensure their successful execution, robust evidence must be generated to show that using antibiotics in the treatment of acne has important implications for future infective episodes and resistance sequelae. Until there is evidence of how the use of oral antibiotics for acne may cause AMR, changing current practice will be challenging.[18]

Given the global health emergency of AMR and the dominant role antibiotics play in the treatment of acne – a highly prevalent and ubiquitous skin condition worldwide, there is a clearly defined evidence gap which needs to be urgently addressed.[19]. This systematic review aims to establish what is already known about resistance sequelae for those with acne who are treated with long-term topical or oral antibiotics.[20]

For peer review only

Methods and Analysis

Literature search strategy

We will search the following databases; Embase, Medline, Cochrane and Web of Science. We will develop search terms by identifying keywords from relevant articles and by undertaking pilot searches to identify index or Mesh terms. We will modify the search terms according to each database e.g. the MeSH terms in Medline and Emtree terms in Embase. Searches will be undertaken by the lead author who has medical and search training in collaboration with a librarian. Search strategies will be reviewed by all authors. The searches will be kept as broad as possible for example, by using the 'explode' function on the Ovid platform to maximise the number of relevant articles. The search strategy is available to view in the accompanying supplement. Searches will be undertaken in July 2019 and will go back to inception of the databases.

Eligibility criteria

Inclusion criteria:

- To address the question, the following inclusion criteria will apply:
  - **Population:** A study population including participants aged over the age of 8 in any healthcare setting.
  - **Intervention:** Oral antibiotics prescribed for acne, for a minimum of 28 days of daily dosing.
  - **Comparison:** People who have not been treated with oral antibiotics for acne (or general population).

For peer review only - http://bmjopen.bmj.com/site/about/guidelines.xhtml

- **Outcome:** Any measure, including proxy measures The primary outcome is antibiotic treatment failure or infection caused by a resistant organism. The secondary outcome is the detection of resistant organisms without an infection, rate of infection or changes to flora. Any measure including proxy measures will be used.
- Original studies will be eligible for assessment for inclusion if they address the specific research question.
- Randomised controlled trials (of any trial design).
- Observational studies limited to cohort and case-control studies.
- We will include conference abstracts if the full paper is unpublished and can be obtained from the authors.

Exclusion criteria:

- Ecological studies and studies that do not assess temporality such as case-series and case reports.
- We will exclude, unpublished studies, ongoing studies and the grey literature.
- In addition studies which only look at antimicrobial resistance to *Propionibacterium acnes* or *P. acnes* or *Cutibacterium acnes C. acnes*).
- Studies including people who are under the age of 8 exclusively will be excluded. The age of 8 was chosen as acne vulgaris is unlikely to present in younger children and in addition, tetracyclines are not recommended in younger children – the BNF recommends tetracyclines are given to children aged 12 years and above.
- Studies including people who are treated with antibiotics for other acne subtypes e.g. hidradenitis suppurativa or drug induced acne.

Exposure

At least 28 days of continuous oral antibiotics for acne, the duration helping ensure treatment is not targeted at an acute infective episode and in addition, 28 days is the minimum duration a prescription will be issued for an antibiotic treatment of acne The exposure is likely to include commonly used antibiotic classes – tetracyclines, macrolides and dihydrofolate reductase inhibitors, however there will be no limits placed on the antibiotic class used to treat acne.

For peer review only - http://bmjopen.bmj.com/site/about/guidelines.xhtml

For peer review only

Comparator

No exposure to long-term oral antibiotics within an acne population or within a general population.

Outcome

The primary outcome is antibiotic treatment failure or infection caused by a resistant organism. The secondary outcome is the detection of resistant organisms without an infection, rate of infection or changes to flora. This will include any measure of AMR, for example, laboratory measures (such as C-reactive Protein or culture), patient observations (such as an elevated temperature and/or pulse rate which may indicate an infective process) or proxy measures that may have been used in epidemiological studies, for example, difficult to treat infections. Each outcome will be assessed separately. The outcome can occur at any time point after at least 28 days of continuous oral antibiotic exposure for acne; we will stratify according to length of follow up, e.g. up to 6 months, 6 month to 1 year, 1-2 years etc.

Potential confounding variables

Confounding factors that may be considered by studies investigating treatment failure or AMR as a result of long-term antibiotics for acne are: age, sex, socioeconomic status, medical conditions such as primary immunodeficiency, diabetes, asthma, cancer requiring immunosuppressive medication, recent hospitalization within the last 6 months, repeated admissions to hospital, any recurrent infections, other prescribed medication in particular immunosuppressive therapy including oral corticosteroids, smoking, alcohol use and ethnicity. The inclusion of these confounding factors will be acknowledged in the bias assessment of each study along with a statement of the direction and magnitude of bias their omission may be associated with.

Eligibility assessment and data extraction

Phase 1: Covidence, an online literature review data management programme will be used to facilitate the systematic review process, inclusive of title and abstract screening, full paper retrieval and storage and decisions on which papers to include at full text review. In the first phase, all titles and abstracts will be uploaded to Covidence. Duplicates will then be removed by the lead reviewer (KB). Three reviewers, KB, LYL and JB will then independently screen the search results based on title and abstract. Each title will require two votes. Consensus will be achieved on the number of titles and abstracts to include in the full study review. Any disputes will be resolved by the involvement of a 4th reviewer, SML.

For peer review only - http://bmjopen.bmj.com/site/about/guidelines.xhtml

For peer review only

Phase 2: Full text papers will be assessed independently by the reviewer pairs using a standardised data extraction form. The extraction tool will be piloted using the first 3 included records, after which modifications may be made following discussion with other members of the review team. The quality of the studies will be scored using assessment tools and free text explanations for the score given will be included on the score sheet and will also be included in supplementary material in the manuscript. Any disagreements will be discussed by the three reviewers (KB, LYL and JB) and in instances of disagreement, a 4th reviewer (SML) will make a final decision. If ambiguity still remains after the full text is obtained, the study authors will be contacted for further clarification.

Data items

Three data domains will be extracted:

Data relating to study design

Author, country, specific study design, the year the study was conducted or the years over which the data were collected. Healthcare setting, the number of study participants, the ages of the participants, the gender balance, and the characteristics and number of comparators, if any. If the study is a trial, then specifics of the study design such as randomisation, allocation concealment and blinding will be noted.

Data relating to exposure

The class of antibiotic used, the median/mean length of treatment of acne with the antibiotic, the definition of long-term treatment with antibiotics used in the study, the number of participants exposed to antibiotics and if multiple courses are prescribed, the length of time between antibiotic courses and the intervention applied to comparators.

Data relating to outcomes

The measure of antibiotic treatment failure or AMR and the degree of antibiotic treatment failure or AMR, e.g. repeat course required, hospitalisation or death. The length of follow up will be stratified.

Study quality assessment

Each study will be critically appraised by reviewers. The Cochrane tool for bias assessment and the ROBINS-I tool for the assessment of bias in non-randomised studies will be used to assess the risk of bias in included studies.[21, 22] GRADE will be used to make an overall assessment of the quality of

For peer review only - http://bmjopen.bmj.com/site/about/guidelines.xhtml

evidence.[1] Pairs of reviewers will make independent assessments of the risk of bias. Markers of bias depending on study design included in the aforementioned scoring tools will include factors such as the method of participant selection, follow up, randomisation, adjustment for confounding and measurement error of exposures or outcomes. If a proportion of studies have a high risk of bias found using the scoring tool, we will do a sensitivity analysis excluding them.

Data synthesis/ statistical analysis

We will analyse interventional and observational studies separately. If there is homogeneity across studies and a meta-analysis is possible, we will generate a pooled effect estimate for those exposed to long-term antibiotics and those unexposed within each category of study design. If there are a sufficient number of studies, subgroup analyses will be undertaken for example, by class of antibiotic and antibiotic treatment duration. The I^2 statistic will be used to assess heterogeneity.[23] Sources of heterogeneity may include methodology, age of participants, study duration, the confounding factors considered, the exposure (i.e. length/duration, the class of antibiotic), the comparators and the outcomes measured. If heterogeneity is above 50% we will not undertake a meta-analysis. If studies are sufficiently homogenous with regard to exposures, comparators and outcomes, a random effects model will be used to generate a pooled relative risk and its 95% confidence interval. Study characteristics and the effect estimate for the association between antibiotics for acne and the specific measure of AMR will be clearly presented. We will also do a sensitivity analysis using a fixed effects model. Publication bias will be assessed using Funnel plots and Egger tests.[24] Forest plots will be presented. All statistical analyses will be performed using Stata. If quantitative synthesis is not possible due to heterogeneity, we will conduct a narrative synthesis. We will also study each category of outcome measure separately: e.g. laboratory based measures of resistance or outcome measures thought to be proxies for AMR using routinely collected health records An overall description of the strength of the body of evidence generated using GRADE will be described.[22]

The study will be reported following PRISMA guidance.[20]

Patient and Public involvement

This systematic review has been informed by the results of the acne Priority Setting Partnership (PSP) (acnepsp.org) in collaboration with the James Lind Alliance (www.jla.nihr.ac.uk). Over 6000 responses were collated and voted upon to give a top 10 list of treatment uncertainties. Two of these top then uncertainties will be addressed with this systematic review:

For peer review only - http://bmjopen.bmj.com/site/about/guidelines.xhtml

1) What is the correct way to use antibiotics in acne to achieve the best outcomes with the least risk?
2) What management strategy should be adopted for the treatment of acne in order to optimise short and long-term outcomes?

In addition, five people comprising members of the public and patients with acne or their carers will attend a focus group to help write the summary which will be used to disseminate the results of this systematic review to the public.

Ethics and dissemination

This systematic review protocol was registered on the 8th of April 2019 on the International Prospective Register of Systematic Reviews (PROSPERO). Any amendments to the protocol will be updated and published on the PROSPERO website with clear notes of where specific changes were made with detailed explanations of why. The results of this systematic review will be submitted for peer-review publication.

References

1. Sterne JA HM, Reeves BCet al. ROBINS-I: a tool for assessing risk of bias in non-randomised studies of interventions. BMJ. 2016 Oct 12;355:i4919.
2. Organization. WH. Global action plan on antimicrobial resistance. . 2015
3. O'Neill J. Tackling Drug-Resistant Infections Globally: Final Report and Recommendations. The review on antimicrobial resistance May 2016
4. Bhate K, Williams HC. Epidemiology of acne vulgaris. The British journal of dermatology. 2013 Mar;168(3):474-85
5. Williams HC, Dellavalle RP, Garner S. Acne vulgaris. Lancet (London, England). 2012 Jan 28;379(9813):361-72
6. Whitehouse HJ FE, El-Mansori I, Layton AM. . Oral antibiotics for acne: are we adopting premium use? Presentation at the Annual Conference of the British Association of Dermatologists, Birmingham, U.K. 5–7 July 2016.
7. Barbieri JS HO, Margolis DJ. Duration of oral tetracycline-class antibiotic therapy and use of topical retinoids for the treatment of acne among general practitioners (GP): A retrospective cohort study. ournal of the American Academy of Dermatology. 2016 Dec;75:1142-50
8. Lee YH LG, Thiboutot DM et al. A retrospective analysis of the duration of oral antibiotic therapy for the treatment of acne among adolescents: Investigating practice gaps and potential cost-savings. Journal of the American Academy of Dermatology. 2014;71
9. Whitehouse H.J. FE E-MIaLAM, . Conference Presentation: Oral antibiotics for acne: are we adopting premium use? (British Association of Dermatologists Annual Conference 2016. 2016
10. National Institute of Health and Care Excellence. Clinical Knowledge Summaries. Acne vulgaris. revised 2014.
11. Zaenglein AL PA, Schlosser BJ. Guidelines of care for the management of acne vulgaris. J Am Acad Dermatol. 2016;74:945-73 e33

For peer review only - http://bmjopen.bmj.com/site/about/guidelines.xhtml

12. Nast A DB, Bettoli V. European evidence-based (S3) guideline for the treatment of acne – update 2016 – short version. Eur Acad Dermatol Venereol. 2016;30:1261-8
13. Barbieri JS JW, Margolis DJ. Trends in prescribing behavior of systemic agents used in the treatment of acne among dermatologists and nondermatologists: A retrospective analysis, 2004-2013. J Am Acad Dermatol. 2017;77:456-63
14. Kuet K.H. FC FE, Eady A,and Layton A.M,. Conference Presentation: A decade later, has the prevalence of skin colonization by resistant propionibacteria increased in our patients with acne? British Association of Dermatologists Annual Conference. 2015
15. Lee SE KJ-M, Jeong SK. Protease-activated receptor-2 mediates the expression of inflammatory cytokines, antimicrobial peptides, and matrix metalloproteinases in keratinocytes in response to Propionibacterium acnes. Arch Dermatol Res. 2010;302:745-56
16. Bienenfeld A, Nagler AR, Orlow SJ. Oral Antibacterial Therapy for Acne Vulgaris: An Evidence-Based Review. American journal of clinical dermatology. 2017 Aug;18(4):469-90
17. Lawes T L-LJ, Nebot CA. Effects of national antibiotic stewardship and infection control strategies on hospital-associated and community-associated meticillin-resistant Staphylococcus aureus infections across a region of Scotland: a non-linear time-series study. Lancet Infect Dis. 2015;15:1438-49
18. Simpson SA WF, Butler CC. General practitioners' perceptions of antimicrobial resistance: a qualitative study. The Journal of antimicrobial chemotherapy. 2007;59:292-6
19. Sinnott SB, K; Margolis, DJ; Langan, SM. Antibiotics and acne: an emerging iceberg of antibiotic resistance? . British Journal of Dermatology 2016;175(6):1127-8
20. http://prisma-statement.org/.
21. Wells GA SB, O'Connell D, et al. . The Newcastle-Ottawa Scale (NOS) for assessing the quality of nonrandomised studies in meta-analyses. . http://wwwohrica/programs/clinical_epidemiology/oxfordasp 2008
22. https://methods.cochrane.org/risk-bias-20-tool.
23. Ioannidis JP PN, Evangelou E. Uncertainty in heterogeneity estimates in meta-analyses. . BMJ. 2007;335:914-6
24. Egger M DSG, Schneider M, Minder C. Bias in meta-analysis detected by a simple, graphical test. BMJ. 1997 315:629-34

For peer review only

BMJ Open

Is there an association between long-term antibiotics for acne and subsequent infection sequelae and antimicrobial resistance? Systematic review protocol

Journal:	*BMJ Open*
Manuscript ID	bmjopen-2019-033662.R1
Article Type:	Protocol
Date Submitted by the Author:	03-Mar-2020
Complete List of Authors:	Bhate, Ketaki; London School of Hygiene and Tropical Medicine Faculty of Epidemiology and Population Health,<br>Lin, Liang-Yu; London School of Hygiene and Tropical Medicine Faculty of Epidemiology and Population Health, Department of Non-communicable Disease Epidemiology;<br>Barbieri, John; University of Pennsylvania, Perelman School of Medicine, Department of Dermatology<br>Leyrat, Clémence; London School of Hygiene and Tropical Medicine, Department of Medical Statistics<br>Hopkins, Susan; Public Health England<br>Stabler, Richard; London School of Hygiene and Tropical Medicine Faculty of Infectious and Tropical Diseases<br>Shallcross, Laura; University College London<br>Smeeth, Liam; London School of Hygiene and Tropical Medicine Faculty of Epidemiology and Population Health<br>Francis, Nick A.; Cardiff University<br>Mathur, Rohini; London School of Hygiene and Tropical Medicine, Department of Non-Communicable Disease Epidemiology; Queen Mary University of London, Centre for Primary Care & Public Health<br>Langan, Sinead; London School of Hygiene and Tropical Medicine<br>Sinnott, Sarah-Jo; London School of Hygiene and Tropical Medicine, Non-communicable disease epidemiology
**Primary Subject Heading**:	Dermatology
Secondary Subject Heading:	Dermatology, Epidemiology
Keywords:	Acne < DERMATOLOGY, antimicrobial resistance, EPIDEMIOLOGY, Dermatological epidemiology < DERMATOLOGY

SCHOLARONE™
Manuscripts

I, the Submitting Author has the right to grant and does grant on behalf of all authors of the Work (as defined in the below author licence), an exclusive licence and/or a non-exclusive licence for contributions from authors who are: i) UK Crown employees; ii) where BMJ has agreed a CC-BY licence shall apply, and/or iii) in accordance with the terms applicable for US Federal Government officers or employees acting as part of their official duties; on a worldwide, perpetual, irrevocable, royalty-free basis to BMJ Publishing Group Ltd ("BMJ") its licensees and where the relevant Journal is co-owned by BMJ to the co-owners of the Journal, to publish the Work in this journal and any other BMJ products and to exploit all rights, as set out in our licence.

The Submitting Author accepts and understands that any supply made under these terms is made by BMJ to the Submitting Author unless you are acting as an employee on behalf of your employer or a postgraduate student of an affiliated institution which is paying any applicable article publishing charge ("APC") for Open Access articles. Where the Submitting Author wishes to make the Work available on an Open Access basis (and intends to pay the relevant APC), the terms of reuse of such Open Access shall be governed by a Creative Commons licence – details of these licences and which Creative Commons licence will apply to this Work are set out in our licence referred to above.

Other than as permitted in any relevant BMJ Author's Self Archiving Policies, I confirm this Work has not been accepted for publication elsewhere, is not being considered for publication elsewhere and does not duplicate material already published. I confirm all authors consent to publication of this Work and authorise the granting of this licence.

For peer review only

BMJ PROTOCOL

Title

Is there an association between long-term antibiotics for acne and subsequent infection sequelae and antimicrobial resistance? Systematic review protocol

Authors

Dr Ketaki Bhate[1] Ketaki.bhate@lshtm.ac.uk https://orcid.org/0000-0001-5509-4428

Dr Liang-Yu Lin[1] liang-yu.lin@lshtm.ac.uk https://orcid.org/0000-0003-4720-6738

Dr John Barbieri[2] john.barbieri@uphs.upenn.edu https://orcid.org/0000-0002-5467-4102

Dr Clemence Leyrat[1] clemence.leyrat@lshtm.ac.uk https://orcid.org/0000-0002-4097-4577

Dr Susan Hopkins[3] susan.hopkins@phe.gov.uk https://orcid.org/0000-0001-5179-5702

Dr Richard Stabler[4] Richard.stabler@lshtm.ac.uk https://orcid.org/0000-0002-2402-6630

Dr Laura Shallcross[5] l.shallcross@ucl.ac.uk https://orcid.org/0000-0003-1713-2555

Professor Liam Smeeth[1] liam.smeeth@lshtm.ac.uk https://orcid.org/0000-0002-9168-6022

Professor Nick Francis[6] francisna@cardiff.ac.uk https://orcid.org/0000-0001-8939-7312

Dr Rohini Mathur[1] rohini.mathur@lshtm.ac.uk https://orcid.org/0000-0002-3817-8790

Professor Sinéad Langan[1*] sinead.langan@lshtm.ac.uk https://orcid.org/0000-0002-7022-7441

Dr Sarah-Jo Sinnott[1*] sarah-jo.sinnott@lshtm.ac.uk https://orcid.org/0000-0001-7586-686X

*Senior authors

Author affiliations

1. Faculty of Epidemiology and Population Health, London School of Hygiene and Tropical Medicine, Keppel Street, London.
2. University of Pennsylvania, Perelman School of Medicine, Philadelphia.
3. AMR Division, Public Health England.
4. Faculty of Infectious and Tropical Diseases, London School of Hygiene and Tropical Medicine, Keppel Street, London.

For peer review only - http://bmjopen.bmj.com/site/about/guidelines.xhtml

5. Faculty of Population Health Sciences, University College London.
6. School of Primary Care, Population Sciences and Medical Education, University of Southampton.

Correspondence to: Dr Ketaki Bhate

London School of Hygiene and Tropical Medicine, Keppel Street, WC1E 7HT

Ketaki.bhate@lshtm.ac.uk

Word count

2060

Keywords

Acne vulgaris, antibiotic, antimicrobial resistance, tetracycline, macrolide, dihydrofolate reductase inhibitor

Author contributions

Ketaki Bhate wrote the protocol. Sinéad Langan, Sarah-Jo Sinnott and Rohini Mathur supervised the writing process and contributed equally. Ling-Yu Lin, John Barbieri, Clemence Leyrat, Richard Stabler, Laura Shallcross, Susan Hopkins, Nick Francis and Liam Smeeth form an advisory group and reviewed the protocol.

Funding statement

Ketaki Bhate is funded by an NIHR Doctoral Research Fellowship DRF-2018-11-ST2-066.

Professor Sinéad Langan is funded by a Wellcome Trust Senior Clinical Fellowship (205039/Z/16/Z).

Dr. Barbieri is supported by the National Institute of Arthritis and Musculoskeletal and Skin Diseases of the National Institutes of Health under award number T32-AR-007465.

Dr Laura Shallcross is funded by an NIHR Clinician Scientist Award CS-2016-16-007

Disclaimer:

For peer review only - http://bmjopen.bmj.com/site/about/guidelines.xhtml

For peer review only

The views expressed are those of the author(s) and not necessarily those of the NHS, the NIHR or the Department of Health and Social Care.

Competing interests

Dr. Barbieri is supported by the National Institute of Arthritis and Musculoskeletal and Skin Diseases of the National Institutes of Health under award number T32-AR-007465 and receives partial salary support through a Pfizer Fellowship grant to the Trustees of the University of Pennsylvania.

For peer review only

ABSTRACT:

Introduction

Antimicrobial resistance (AMR) is a global health emergency. Acne vulgaris is a highly prevalent condition, and the dominant role antibiotics play in its treatment is a major concern. Antibiotics are widely used in the treatment of acne predominantly for their anti-inflammatory effect, hence their use in acne may not be optimal. Tetracyclines and macrolides are the two most common oral antibiotics classes prescribed, and average use can extend from –a few months to several years of intermittent or continuous use. This overall aim of this systematic review is to elucidate what is known about oral antibiotics for acne contributing to antibiotic treatment failure and AMR.

Methods and Analysis

A systematic review will be conducted to address the question: What is the existing evidence that long-term oral antibiotics used to treat acne in those over 8 years of age contribute towards antibiotic treatment failure or other outcomes suggestive of the impact of AMR? We will search the following databases: Embase, MEDLINE, the Cochrane library and Web of Science. Search terms will be developed in collaboration with a librarian by identifying keywords from relevant articles and by undertaking pilot searches. Randomised-control trials, cohort and case-controlled studies conducted in any health care setting and published in any language will be included. The searches will be re-run prior to final analyses to capture the recent literature. The Cochrane tool for bias assessment in randomised trials and ROBINS-I for the assessment of bias in non-randomised studies will be used to assess the risk of bias of included studies. GRADE will be used to make an overall assessment of the quality of evidence. A meta-analysis will be undertaken of the outcome measures if the individual studies are sufficiently homogenous. If a meta-analysis is not possible, a qualitative assessment will be presented as a narrative review.

Ethics and dissemination

Ethical approval is not required for this systematic-review. The results will be published in a peer-reviewed journal and any deviations from the protocol will be clearly documented in the published manuscript of the full systematic-review.

Prospero registration number

CRD42019121738.

For peer review only

Strengths and limitations of this study

- To our knowledge, this is the first comprehensive systematic review that will address the use of oral antibiotics for acne and their contribution to antimicrobial resistance
- Screening, data extraction and quality assessment will be undertaken independently by three medically qualified researchers with training in systematic review methodology, thereby ensuring scientific rigour, transparency and repeatability
- There are no date or language restrictions; however, this systematic review does not examine the grey literature

For peer review only

For peer review only - http://bmjopen.bmj.com/site/about/guidelines.xhtml

Introduction

The future effectiveness of antibiotics is in jeopardy with the World Health Organisation declaring the threat of Antimicrobial Resistance (AMR) a most urgent crisis. [1] Future deaths from infections as a result of AMR without any intervention is estimated at 10 million per year and by 2050, the cost of AMR could reach 100 trillion USD.[2]

Topical and oral antibiotics are commonly prescribed for the treatment of acne vulgaris, a chronic skin disorder with onset predominantly in adolescence. Given the psychosocial consequences and potential for permanent disfigurement with scarring, it is imperative that people with acne receive effective treatment.[3, 4] Prevalence studies show that 80-100% of teenagers have acne and that 20% are moderately to severely affected. The high prevalence means that both topical and oral antibiotics are used in a large proportion of the adolescent population and for variable durations ranging from 6 weeks to many months, and in some cases, several years.[5, 6] Differences between international guidelines regarding duration of treatment is one of the reasons that antibiotics for acne are used for significantly longer than recommended as there is uncertainty about the optimal duration of treatment.[6-11] Tetracyclines and macrolides are the two of the most common oral antibiotic classes prescribed for acne with varying durations of average use depending on treatment setting and between different countries.[6, 12]

The overuse of antibiotics is a known cause of AMR as repeated and sustained exposure allows microbes to develop mechanisms to avoid the effects of the drugs designed to treat them and allows selection in favour of bystander or commensal bacteria with resistance subsequently cause invasive infection. Acne is aetiologically is multifactorial, we already know that some strains of *Cutibacterium acnes* (formally *Propionibacterium acnes*), the bacteria pathophysiologically associated with acne, are now resistant to commonly used antibiotics in acne, making their initial use as anti-microbial agents futile.[13, 14] However, we do not know how these long-term antibiotics for acne may attenuate microbiota elsewhere at other body sites, and the ability of other bacteria at other infective sites to withstand the effect of antibiotics. Despite this, the anti-inflammatory effect and proven efficacy of antibiotics in treating acne ensures their continued use[15], albeit their effects may not be sustained. Considering the relationship between long term exposure to antibiotics and AMR, this practice may not be optimal.

The effects long-term antibiotics for acne have on future infections caused by resistant organisms, subsequent antibiotic treatment failure or the rate of infections (or any other measures which may indicate antimicrobial resistance) and how long any effect may last, is not yet known and has not been systematically reviewed in the literature before. While antibiotic stewardship programmes

For peer review only - http://bmjopen.bmj.com/site/about/guidelines.xhtml

have been shown to be effective [16] in other settings, to ensure their successful execution, robust evidence must be generated to show that using antibiotics in the treatment of acne has important implications for future infective episodes and resistance sequelae. Until there is evidence of how the use of oral antibiotics for acne may cause AMR, changing current practice will be challenging.[17]

Given the global health emergency of AMR and the dominant role antibiotics play in the treatment of acne – a highly prevalent and ubiquitous skin condition worldwide, there is a clearly defined evidence gap which needs to be urgently addressed.[18]. This systematic review aims to establish what is already known about resistance sequelae for those with acne who are treated with long-term topical or oral antibiotics.[19]

For peer review only

Methods and Analysis

Literature search strategy

We will search the following databases; Embase, Medline, Cochrane and Web of Science. We will develop search terms by identifying keywords from relevant articles and by undertaking pilot searches to identify index or Mesh terms. We will modify the search terms according to each database e.g. the MeSH terms in Medline and Emtree terms in Embase. Searches will be undertaken by the lead author who has medical and search training in collaboration with a librarian. Search strategies will be reviewed by all authors. The searches will be kept as broad as possible for example, by using the 'explode' function on the Ovid platform to maximise the number of relevant articles. The search strategy is available to view in the accompanying supplement (supplementary file 1). Searches were undertaken on the 19th of July 2019 and date back to inception of the databases.

Eligibility criteria

Inclusion criteria:

- To address the question, the following inclusion criteria will apply:
    - **Population:** A study population including participants aged over the age of 8 in any healthcare setting with acne vulgaris.
- Original studies will be eligible for assessment for inclusion if they address the specific research question.
- Randomised controlled trials (of any trial design).
- Observational studies limited to cohort and case-control studies.

For peer review only - http://bmjopen.bmj.com/site/about/guidelines.xhtml

- We will include conference abstracts if the full paper is unpublished and can be obtained from the authors.

Exclusion criteria:

- Ecological studies and studies that do not assess temporality such as case-series and case reports.
- We will exclude, unpublished studies, ongoing studies and the grey literature.
- In addition studies which only look at antimicrobial resistance in *Propionibacterium acnes* or *P. acnes* or *Cutibacterium acnes C. acnes*).
- Studies including people who are under the age of 8 exclusively will be excluded. The age of 8 was chosen as acne vulgaris is unlikely to present in younger children and in addition, tetracyclines are not recommended in younger children – the BNF recommends tetracyclines are given to children aged 12 years and above.
- Studies including people who are treated with antibiotics for other acne subtypes e.g. hidradenitis suppurativa or drug induced acne.

Exposure

At least 28 days of continuous (daily doses) oral antibiotics for acne vulgaris, the duration helping ensure treatment is not targeted at an acute infective episode and in addition, 28 days is the minimum duration a prescription will be issued for an antibiotic treatment of acne. The exposure is likely to include commonly used antibiotic classes – tetracyclines, macrolides and dihydrofolate reductase inhibitors, however there will be no limits placed on the antibiotic class used to treat acne. We have excluded the use of topical antibiotics are these are less likely to have an effect at sites other than the skin to where they are applied.

Comparator

No exposure to long-term oral antibiotics within an acne population or within a general population.

Outcome

The primary outcome is antibiotic treatment failure or any infection caused by a resistant organism. The secondary outcome is the detection of resistant organisms without a clinical infection, rate of

For peer review only - http://bmjopen.bmj.com/site/about/guidelines.xhtml

infection or changes to the microbiota profile e.g. with the colonisation of resistant microbiota without a clinical infection, or different microbiota in a sampled site compared to baseline prior to having received a long-term antibiotic for acne. Any measure (including proxy measures) will be included, for example, laboratory measures (such as an elevated C-reactive Protein or positive culture in the case of an infection at any body site), patient observations (such as an elevated temperature and/or pulse rate which may indicate an infective process) or proxy measures that may have been used in epidemiological studies, for example, difficult to treat infections which may indicate a resistant infection. Each outcome will be assessed separately. The outcome can occur at any time point after at least 28 days of continuous oral antibiotic exposure for acne; we will stratify according to length of follow up, e.g. up to 6 months, 6 months to 1 year, 1-2 years etc.

Potential confounding variables/ effect modifiers

Confounding factors that may be considered by studies investigating treatment failure or AMR as a result of long-term antibiotics for acne are: age, sex, socioeconomic status, treatment adherence, medical conditions such as primary immunodeficiency, diabetes, asthma, cancer requiring immunosuppressive medication, recent hospitalization within the last 6 months, repeated admissions to hospital, any recurrent infections, other prescribed medication in particular immunosuppressive therapy including oral corticosteroids, smoking, alcohol use and ethnicity. We will also explore for effect modification. The inclusion of these confounding factors will be acknowledged in the bias assessment of each study along with a statement of the direction and magnitude of bias their omission may be associated with.

Eligibility assessment and data extraction

Phase 1: Covidence, an online literature review data management programme will be used to facilitate the systematic review process, inclusive of title and abstract screening, full paper retrieval and storage and decisions on which papers to include at full text review. In the first phase, all titles and abstracts will be uploaded to Covidence. Duplicates will then be removed by the lead reviewer (KB). Three reviewers, KB, LYL and JB will then independently screen the search results based on title and abstract. Each title/abstract will require two votes. Consensus will be achieved on the number of titles and abstracts to include in the full study review. Any disputes will be resolved by the involvement of a 4th reviewer, SML.

Phase 2: Full text papers will be assessed independently by the reviewer pairs using a standardised data extraction form. The extraction tool will be piloted using the first 3 included records, after

For peer review only - http://bmjopen.bmj.com/site/about/guidelines.xhtml

For peer review only

which modifications may be made following discussion with other members of the review team. The quality of the studies will be scored using assessment tools and free text explanations for the score given will be included on the score sheet. Any disagreements will be discussed by the three reviewers (KB, LYL and JB) and in instances of disagreement, a 4th reviewer (SML) will make a final decision. If ambiguity still remains after the full text is obtained, the study authors will be contacted for further clarification.

Data items

Three data domains will be extracted:

Data relating to study design

Author, country, specific study design, the year the study was conducted or the years over which the data were collected. Healthcare setting, the number of study participants, the ages of the participants, the gender balance will be collected for the whole population under study, including the comparator group. If the study is a trial, then specifics of the study design such as randomisation, allocation concealment and blinding will be noted.

Data relating to exposure

The dose, frequency and antibiotic used, the median/mean length of treatment of acne with the antibiotic, the definition of long-term treatment with antibiotics used in the study, the number of participants exposed to antibiotics and if multiple courses are prescribed, the length of time between antibiotic courses and the intervention applied to comparators.

Data relating to outcomes

The measure of antibiotic treatment failure or AMR and the degree of antibiotic treatment failure or AMR, e.g. repeat course required, hospitalisation or death. The length of follow up will be stratified.

Study quality assessment

Each study will be critically appraised by reviewers. The Cochrane tool for bias assessment in randomised studies and the ROBINS-I tool for the assessment of bias in non-randomised studies will be used to assess the risk of bias in included studies.[20-22] GRADE will be used to make an overall assessment of the quality of evidence.[22] Pairs of reviewers will make independent assessments of the risk of bias. Markers of bias depending on study design included in the aforementioned scoring

For peer review only - http://bmjopen.bmj.com/site/about/guidelines.xhtml

tools will include factors such as the method of participant selection, follow up, randomisation, adjustment for confounding and measurement error of exposures or outcomes. If a proportion of studies have a high risk of bias found using the scoring tool, we will do a sensitivity analysis excluding them.

Data synthesis/ statistical analysis

We will analyse interventional and observational studies separately. If there is homogeneity across studies and a meta-analysis is possible, we will generate a pooled effect estimate for those exposed to long-term antibiotics and those unexposed within each category of study design. If there are a sufficient number of studies, subgroup analyses will be undertaken for example, by class of antibiotic and antibiotic treatment duration. The I^2 statistic will be used to assess heterogeneity.[23] Sources of heterogeneity may include methodology, age of participants, study duration, the confounding factors considered, the exposure (i.e. length/duration, the class of antibiotic), the comparators and the outcomes measured. If heterogeneity is above 50% we will not undertake a meta-analysis. If studies are sufficiently homogenous with regard to exposures, comparators and outcomes, a random effects model will be used to generate a pooled relative risk and its 95% confidence interval. Study characteristics and the effect estimate for the association between antibiotics for acne and the specific measure of AMR will be clearly presented. We will also do a sensitivity analysis using a fixed effects model. Publication bias will be assessed using Funnel plots and Egger tests.[24] Forest plots will be presented. All statistical analyses will be performed using Stata. If quantitative synthesis is not possible due to heterogeneity, we will conduct a narrative synthesis. We will also study each category of outcome measure separately: e.g. laboratory-based measures of resistance or outcome measures thought to be proxies for AMR using routinely collected health records. Given the breadth of outlined outcomes, it is likely that the evidence obtained will be diverse. An overall description of the strength of the body of evidence generated using GRADE will be described.[21]

The study will be reported following PRISMA guidance.[19]

Patient and Public involvement

This systematic review has been informed by the results of the acne Priority Setting Partnership (PSP) (acnepsp.org) in collaboration with the James Lind Alliance (www.jla.nihr.ac.uk). Over 6000 responses were collated and voted upon to give a top 10 list of treatment uncertainties. Two of these top then uncertainties will be addressed with this systematic review:

For peer review only

For peer review only - http://bmjopen.bmj.com/site/about/guidelines.xhtml

1) What is the correct way to use antibiotics in acne to achieve the best outcomes with the least risk?
2) What management strategy should be adopted for the treatment of acne in order to optimise short and long-term outcomes?

In addition, five people comprising members of the public and patients with acne or their carers will attend a focus group to help write the summary which will be used to disseminate the results of this systematic review to the public.

Ethics and dissemination

As this is a systematic review, ethical approval was not required. This systematic review protocol was registered on the 8th of April 2019 on the International Prospective Register of Systematic Reviews (PROSPERO). Any amendments to the protocol will be updated and published on the PROSPERO website with clear notes of where specific changes were made with detailed explanations of why. The results of this systematic review will be submitted for peer-review publication.

References

1. World Health Organization. Global action plan on antimicrobial resistance. 2015
2. O'Neill J. Tackling Drug-Resistant Infections Globally: Final Report and Recommendations. The review on antimicrobial resistance May 2016
3. Bhate K, Williams HC. Epidemiology of acne vulgaris. The British journal of dermatology. 2013 Mar;168(3):474-85
4. Williams HC, Dellavalle RP, Garner S. Acne vulgaris. Lancet (London, England). 2012 Jan 28;379(9813):361-72
5. Whitehouse HJ FE, El-Mansori I, Layton AM. . Oral antibiotics for acne: are we adopting premium use? Presentation at the Annual Conference of the British Association of Dermatologists, Birmingham, U.K. 5–7 July 2016.
6. Barbieri JS HO, Margolis DJ. Duration of oral tetracycline-class antibiotic therapy and use of topical retinoids for the treatment of acne among general practitioners (GP): A retrospective cohort study. ournal of the American Academy of Dermatology. 2016 Dec;75:1142-50
7. Lee YH LG, Thiboutot DM et al. A retrospective analysis of the duration of oral antibiotic therapy for the treatment of acne among adolescents: Investigating practice gaps and potential cost-savings. Journal of the American Academy of Dermatology. 2014;71
8. Whitehouse H.J. FE E-MIaLAM, . Conference Presentation: Oral antibiotics for acne: are we adopting premium use? (British Association of Dermatologists Annual Conference 2016. 2016
9. National Institute of Health and Care Excellence. Clinical Knowledge Summaries. Acne vulgaris. revised 2014.
10. Zaenglein AL PA, Schlosser BJ. Guidelines of care for the management of acne vulgaris. J Am Acad Dermatol. 2016;74:945-73 e33
11. Nast A DB, Bettoli V. European evidence-based (S3) guideline for the treatment of acne – update 2016 – short version. Eur Acad Dermatol Venereol. 2016;30:1261-8

For peer review only - http://bmjopen.bmj.com/site/about/guidelines.xhtml

12. Barbieri JS JW, Margolis DJ. Trends in prescribing behavior of systemic agents used in the treatment of acne among dermatologists and nondermatologists: A retrospective analysis, 2004-2013. J Am Acad Dermatol. 2017;77:456-63
13. Kuet K.H. FC FE, Eady A,and Layton A.M,. Conference Presentation: A decade later, has the prevalence of skin colonization by resistant propionibacteria increased in our patients with acne? British Association of Dermatologists Annual Conference. 2015
14. Lee SE KJ-M, Jeong SK. Protease-activated receptor-2 mediates the expression of inflammatory cytokines, antimicrobial peptides, and matrix metalloproteinases in keratinocytes in response to Propionibacterium acnes. Arch Dermatol Res. 2010;302:745-56
15. Bienenfeld A, Nagler AR, Orlow SJ. Oral Antibacterial Therapy for Acne Vulgaris: An Evidence-Based Review. American journal of clinical dermatology. 2017 Aug;18(4):469-90
16. Lawes T L-LJ, Nebot CA. Effects of national antibiotic stewardship and infection control strategies on hospital-associated and community-associated meticillin-resistant Staphylococcus aureus infections across a region of Scotland: a non-linear time-series study. Lancet Infect Dis. 2015;15:1438-49
17. Simpson SA WF, Butler CC. General practitioners' perceptions of antimicrobial resistance: a qualitative study. The Journal of antimicrobial chemotherapy. 2007;59:292-6
18. Sinnott SB, K; Margolis, DJ; Langan, SM. Antibiotics and acne: an emerging iceberg of antibiotic resistance? . British Journal of Dermatology 2016;175(6):1127-8
19. http://prisma-statement.org/.
20. Wells GA SB, O'Connell D, et al. . The Newcastle-Ottawa Scale (NOS) for assessing the quality of nonrandomised studies in meta-analyses. . http://wwwohrica/programs/clinical_epidemiology/oxfordasp 2008
21. https://methods.cochrane.org/risk-bias-20-tool.
22. Sterne JA HM, Reeves BCet al. ROBINS-I: a tool for assessing risk of bias in non-randomised studies of interventions. BMJ. 2016 Oct 12;355:i4919.
23. Ioannidis JP PN, Evangelou E. Uncertainty in heterogeneity estimates in meta-analyses. . BMJ. 2007;335:914-6
24. Egger M DSG, Schneider M, Minder C. Bias in meta-analysis detected by a simple, graphical test. BMJ. 1997 315:629-34

For peer review only

Database: Ovid MEDLINE(R) and Epub Ahead of Print, In-Process & Other Non-Indexed Citations, Daily and Versions(R) <1946 to June 21, 2019>
Search Strategy:
----
1 acne.mp. (17491)
2 exp Acne Vulgaris/ (11259)
3 1 or 2 (17491)
4 antibiotic*.mp. (355427)
5 exp Antibiotic Prophylaxis/ (13110)
6 exp Anti-Bacterial Agents/ (700080)
7 tetracycline*.mp. (45045)
8 exp Tetracycline/ (19631)
9 exp Tetracyclines/ (46884)
10 lymecycline*.mp. (168)
11 exp Lymecycline/ (119)
12 minocycline*.mp. (8527)
13 exp Minocycline/ (5724)
14 doxycycline*.mp. (16071)
15 exp Doxycycline/ (9287)
16 oxytetracycline*.mp. (8262)
17 exp Oxytetracycline/ (6279)
18 macrolide*.mp. (22555)
19 Macrolides/ (11795)
20 exp Erythromycin/ (24397)
21 erythromycin*.mp. (25510)
22 clarithromycin*.mp. (10167)
23 exp Clarithromycin/ (6062)
24 azithromycin*.mp. (8538)
25 exp Azithromycin/ (4820)
26 dihydrofolate reductase inhibitor*.mp. (346)
27 exp Folic Acid Antagonists/ (57013)
28 trimethoprim*.mp. (21485)
29 exp Trimethoprim/ (11693)
30 exp Trimethoprim, Sulfamethoxazole Drug Combination/ (6696)
31 penicillin*.mp. (82869)
32 exp Penicillin-Binding Proteins/ (3293)
33 exp Penicillin G/ (38077)
34 cephalosporin*.mp. (32358)
35 exp Cephalosporins/ (41273)
36 exp beta-Lactamases/ (22172)
37 fluoroquinolone*.mp. (22199)
38 exp Fluoroquinolones/ (31393)
39 exp Ciprofloxacin/ (12824)
40 aminoglycoside*.mp. (23235)
41 exp Aminoglycosides/ (151256)
42 exp Gentamicins/ (18634)
43 antimicrobial*.mp. (154537)
44 exp Antimicrobial Stewardship/ (725)
45 exp Disk Diffusion Antimicrobial Tests/ (1536)
46 4 or 5 or 6 or 7 or 8 or 9 or 10 or 11 or 12 or 13 or 14 or 15 or 16 or 17 or 18 or 19 or 20

For peer review only

or 21 or 22 or 23 or 24 or 25 or 26 or 27 or 28 or 29 or 30 or 31 or 32 or 33 or 34 or 35 or 36 or 37 or 38 or 39 or 40 or 41 or 42 or 43 or 44 or 45 (1080981)
47 resistance*.mp. (827828)
48 exp beta-Lactam Resistance/ (26155)
49 exp Drug Resistance, Microbial/ or exp Microbial Sensitivity Tests/ (231349)
50 exp Drug Resistance, Multiple/ (33795)
51 exp Drug Resistance, Bacterial/ (83040)
52 exp Methicillin Resistance/ (10188)
53 exp Multidrug Resistance-Associated Proteins/ (14320)
54 exp Vancomycin Resistance/ (3263)
55 47 or 48 or 49 or 50 or 51 or 52 or 53 or 54 (900383)
56 43 or 44 [antimicrobial altogether] (154537)
57 55 and 56 [antimicrobial AND resistance] (70921)
58 46 and 55 [antibiotic AND resistance] (248811)
59 infect*.mp. (2131927)
60 exp Escherichia coli/ (270735)
61 exp Bacteriophages/ (56525)
62 exp Infection/ (760393)
63 infection*.mp. (1804659)
64 59 or 60 or 61 or 62 [infection altogether] (2649927)
65 55 or 57 or 58 [resistance OR antimicrobial resistance OR antibiotic resistance] (900383)
66 64 or 65 [infection OR resistance altogether] (3306493)
67 3 and 66 [combined with acne] (3142)

For peer review only

PRISMA-P 2015 Checklist

This checklist has been adapted for use with protocol submissions to *Systematic Reviews* from Table 3 in Moher D et al: Preferred reporting items for systematic review and meta-analysis protocols (PRISMA-P) 2015 statement. *Systematic Reviews* 2015 **4**:1

Section/topic	#	Checklist item	Information reported		Line number(s)
			Yes	No	
**ADMINISTRATIVE INFORMATION**					
**Title**					
Identification	1a	Identify the report as a protocol of a systematic review	☒	☐	
Update	1b	If the protocol is for an update of a previous systematic review, identify as such	☐	☐	
**Registration**	2	If registered, provide the name of the registry (e.g., PROSPERO) and registration number in the Abstract	☒	☐	
**Authors**					
Contact	3a	Provide name, institutional affiliation, and e-mail address of all protocol authors; provide physical mailing address of corresponding author	☒	☐	
Contributions	3b	Describe contributions of protocol authors and identify the guarantor of the review	☒	☐	
**Amendments**	4	If the protocol represents an amendment of a previously completed or published protocol, identify as such and list changes; otherwise, state plan for documenting important protocol amendments	☒	☐	
**Support**					
Sources	5a	Indicate sources of financial or other support for the review	☒	☐	
Sponsor	5b	Provide name for the review funder and/or sponsor	☒	☐	
Role of sponsor/funder	5c	Describe roles of funder(s), sponsor(s), and/or institution(s), if any, in developing the protocol	☒	☐	
**INTRODUCTION**					
**Rationale**	6	Describe the rationale for the review in the context of what is already known	☒	☐	
**Objectives**	7	Provide an explicit statement of the question(s) the review will address with reference to participants, interventions, comparators, and outcomes (PICO)	☒	☐	

BioMed Central The Open Access Publisher

Section/topic	#	Checklist item	Information reported		Line number(s)
			Yes	No	
**METHODS**					
**Eligibility criteria**	8	Specify the study characteristics (e.g., PICO, study design, setting, time frame) and report characteristics (e.g., years considered, language, publication status) to be used as criteria for eligibility for the review	☒	☐	
**Information sources**	9	Describe all intended information sources (e.g., electronic databases, contact with study authors, trial registers, or other grey literature sources) with planned dates of coverage	☒	☐	
**Search strategy**	10	Present draft of search strategy to be used for at least one electronic database, including planned limits, such that it could be repeated	☒	☐	
STUDY RECORDS					
Data management	11a	Describe the mechanism(s) that will be used to manage records and data throughout the review	☒	☐	
Selection process	11b	State the process that will be used for selecting studies (e.g., two independent reviewers) through each phase of the review (i.e., screening, eligibility, and inclusion in meta-analysis)	☒	☐	
Data collection process	11c	Describe planned method of extracting data from reports (e.g., piloting forms, done independently, in duplicate), any processes for obtaining and confirming data from investigators	☒	☐	
**Data items**	12	List and define all variables for which data will be sought (e.g., PICO items, funding sources), any pre-planned data assumptions and simplifications	☒	☐	
**Outcomes and prioritization**	13	List and define all outcomes for which data will be sought, including prioritization of main and additional outcomes, with rationale	☒	☐	
**Risk of bias in individual studies**	14	Describe anticipated methods for assessing risk of bias of individual studies, including whether this will be done at the outcome or study level, or both; state how this information will be used in data synthesis	☒	☐	
DATA					
**Synthesis**	15a	Describe criteria under which study data will be quantitatively synthesized	☒	☐	
	15b	If data are appropriate for quantitative synthesis, describe planned summary measures, methods of handling data, and methods of combining data from studies, including any planned exploration of consistency (e.g., I^2, Kendall's tau)	☒	☐	
	15c	Describe any proposed additional analyses (e.g., sensitivity or subgroup analyses, meta-regression)	☒	☐	
	15d	If quantitative synthesis is not appropriate, describe the type of summary planned	☒	☐	
**Meta-bias(es)**	16	Specify any planned assessment of meta-bias(es) (e.g., publication bias across studies, selective	☐	☐	

For peer review only

3

Section/topic	#	Checklist item	Information reported		Line number(s)
			Yes	No	
		reporting within studies)			
**Confidence in cumulative evidence**	17	Describe how the strength of the body of evidence will be assessed (e.g., GRADE)	☒	☐	

For peer review only

BioMed Central The Open Access Publisher
